# Molecular Ballet: Investigating the Complex Interaction between Self-Assembling Dendrimers and Human Serum Albumin via Computational and Experimental Methods

**DOI:** 10.3390/pharmaceutics16040533

**Published:** 2024-04-12

**Authors:** Gabriele Cavalieri, Domenico Marson, Nicoletta Giurgevich, Rachele Valeri, Fulvia Felluga, Erik Laurini, Sabrina Pricl

**Affiliations:** 1 Molecular Biology and Nanotechnology Laboratory (MolBNL@UniTS), DEA, University of Trieste, Piazzale Europa 1, 34127 Trieste, Italy; gabriele.cavalieri@phd.units.it (G.C.); domenico.marson@dia.units.it (D.M.); nicoletta.giurgevich@studenti.units.it (N.G.); rachela_valeri@yahoo.it (R.V.); sabrina.pricl@dia.units.it (S.P.); 2 Department of Chemical and Pharmaceutical Sciences, DSCF, University of Trieste, Via Giorgieri 1, 34127 Trieste, Italy; ffelluga@units.it; 3 Department of General Biophysics, Faculty of Biology and Environmental Protection, University of Lodz, ul. Pomorska 141/143, 90-236 Łódź, Poland

**Keywords:** human serum albumin, amphiphilic dendrimer, isothermal titration calorimetry, spectroscopy, HPC molecular simulations

## Abstract

Dendrimers, intricate macromolecules with highly branched nanostructures, offer unique attributes including precise control over size, shape, and functionality, making them promising candidates for a wide range of biomedical applications. The exploration of their interaction with biological environments, particularly human serum albumin (HSA), holds significant importance for biomedical utilization. In this study, the interaction between HSA and a recently developed self-assembling amphiphilic dendrimer (AD) was investigated using various experimental techniques. Fluorescence spectroscopy and isothermal titration calorimetry revealed moderate interactions between the protein and the AD nanomicelles (NMs), primarily attributed to favorable enthalpic contributions arising from electrostatic interactions and hydrogen bonding. Structural analysis indicated minimal changes in HSA upon complexation with the AD NMs, which was further supported by computational simulations demonstrating stable interactions at the atomistic level. These findings provide valuable insights into the binding mechanisms and thermodynamic parameters governing HSA/AD NM interactions, thereby contributing to the understanding of their potential biomedical applications.

## 1. Introduction

Dendrimers represent a class of highly branched macromolecules with unique nanostructures. Their distinctive architecture, characterized by successive layers of branching emanating from a central core, imparts remarkable chemical and physical properties, distinguishing them from traditional linear polymers. Unlike linear polymers, dendrimers exhibit well-defined and uniform structures, resembling miniature trees with precise control over their size, shape, and functionality. This precise architecture grants dendrimers numerous advantages, including high surface-to-volume ratios, monodispersity, and the ability to encapsulate guest molecules within their internal void spaces. The structure of dendrimers typically comprises three fundamental components: a central core featuring multiple reactive sites, branches that extend from the core in a radially symmetric manner, and terminal functional groups decorating the outermost layer. This dendritic topology enables dendrimers to possess a high degree of molecular uniformity, with each generation exhibiting a defined number of branches emanating from the core. Furthermore, the stepwise synthetic approach employed in dendrimer synthesis facilitates precise control over the size and functionalization of dendrimers, offering unparalleled versatility in tailoring their properties for specific applications [1,2,3,4].

The unique characteristics of dendrimers have led to their widespread exploration and utilization across various scientific disciplines and industrial sectors. In particular, dendrimers have found extensive applications in fields such as drug delivery, gene therapy, diagnostics, materials science, and nanotechnology [5,6]. Their ability to encapsulate and deliver therapeutic agents with precision and efficiency has positioned dendrimers as promising candidates for the development of next-generation drug delivery systems. Additionally, their tunable surface chemistry enables the design of dendrimer-based materials for targeted imaging, sensing, and diagnostic applications [7,8].

The advantages associated with dendrimers employed in medical and healthcare applications primarily fall within the purview of therapeutic delivery. Leveraging tailored drug delivery systems effectively mitigates concerns inherent to free drug administration. These concerns include short half-lives, aqueous insolubility, aberrations in biodistribution, and pharmacokinetic deviations of the administered therapeutics. The precise targeting of different anatomical locales or tissue types serves to curtail toxicity, uphold therapeutic thresholds through controlled release kinetics, and enhance therapy bioavailability by preventing premature degradation and increasing absorptive efficiency, exemplifying the realm of controlled drug delivery [9].

Among the most extensively explored dendrimer families is the poly(amidoamine) (PAMAM) dendrimer. However, their translation into clinical settings requires the ready availability of high-quality consistent quantities. Regrettably, the iterative nature of dendrimer synthesis poses significant challenges, with structural anomalies escalating with generation number. These defective dendrimers closely resemble their intact counterparts in chemical composition and physical attributes, complicating purification endeavors. This crucial limitation contributed to the failure of a preclinical trial involving dendrimer-mediated delivery of an anticancer agent [10,11].

To address these challenges, recent emphasis has shifted toward the self-assembly strategy for constructing noncovalent supramolecular dendrimers. Small amphiphilic dendrimers featuring hydrophobic alkyl chains and hydrophilic PAMAM dendrons offer a promising avenue. Synthesis of these molecules in large quantities with high purity is achievable through divergent, convergent, or hybrid methodologies. Taking advantage of their amphipathic character, amphiphilic dendrimers exploit hydrophobic interactions in their core and hydrogen bonds within dendron shells to spontaneously form stable supramolecular dendrimers. Tailoring the length and composition of hydrophobic chains and PAMAM dendron generations, alongside modifying terminal functionalities, imparts unparalleled structural versatility for diverse biomedical applications.

**Figure 1:**
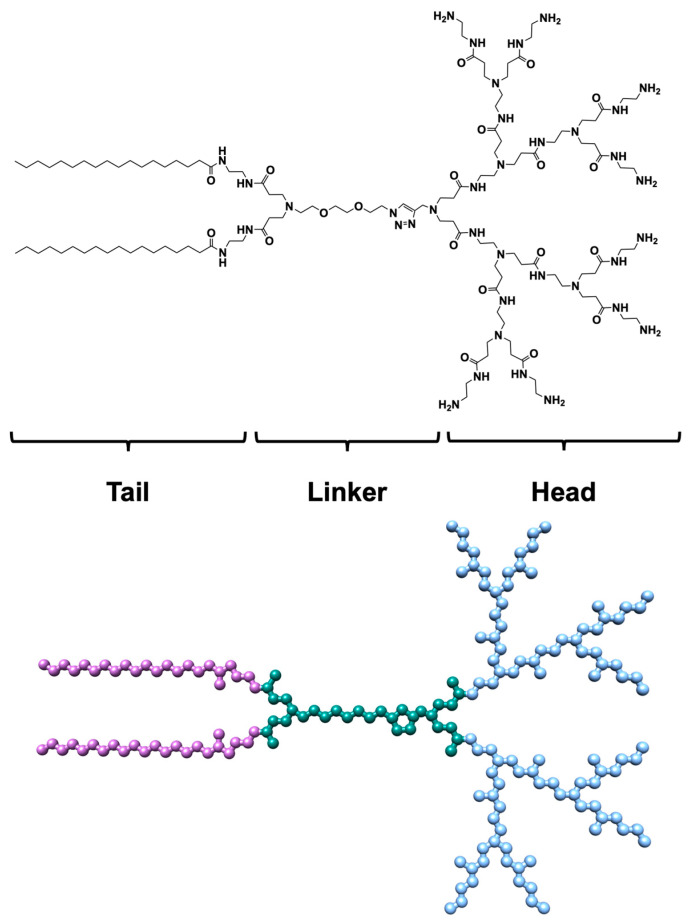
(**Upper panel**) 2D chemical structure of the AD monomer. (**Lower panel**) Atomistic representation of the AD monomer. The atoms are represented as spheres and colored by the corresponding region as follows: hydrophobic tails, purple; linker, teal; polar head, sky blue. For clarity, hydrogen atoms are missing.

Among the myriad of newly engineered and synthesized molecules capable of self-assembly, our team has recently proposed and investigated the amphiphilic dendrimer (AD). Comprising two lipid-like C_18_ chains and a PAMAM dendron head (Figure 1), this dendrimer spontaneously organizes into nearly spherical nanomicelles (NMs), characterized by a micellar diameter of approximately 10 nm, a critical micellar concentration (CMC) ranging from 3.2 to 5.7 µM, and an aggregation number (N_agg_) of 17 AD monomers per micelle, as determined through small-angle X-ray scattering (SAXS), nuclear magnetic resonance (NMR), and isothermal titration calorimetry (ITC). These AD NMs have shown promise as nanovectors (NVs) for gene therapy [12] and the delivery of poorly soluble antitumoral drugs [13]. For example, in a recent study, AD NMs were used to encapsulate Vemurafenib (VEM) and Dabrafenib (DAB), two clinically used anticancer drugs for melanoma, aiming to mitigate major side effects and enhance drug effectiveness. This led to the formation of well-defined core–shell NMs (with high encapsulation efficiency (∼70% for DAB and ∼60% for VEM) and drug loading capacity (∼27% and ∼24% for DAB and VEM, respectively). Importantly, in vitro testing of AD NMs loaded with DAB and VEM in four melanoma cell lines revealed enhanced responses compared to free drug treatment.

An important issue common to all NVs arises from their uncertain behavior in vivo due to the emergence of a protein corona (PC) upon interaction with biological settings [14,15,16,17]. When these systems encounter biological fluids, they promptly acquire a layer of biomolecules, predominantly proteins, leading to PC formation. This protein coat surrounding the NVs poses significant challenges for the biomedical application of nanomaterials, particularly regarding biodistribution and toxicity. Today, the connection between the surface attributes of the NVs and the characteristics/composition of the PC remains elusive. For example, studies indicate that NV traits such as surface charge, chemical nature of the terminal groups, and size (among others) significantly influence the nature of the protein corona. Additionally, PC molecular attributes and composition do affect the cellular uptake of the NVs; for instance, while, occasionally, the PC acts as a barrier, protecting the NVs and limiting their direct interaction with biological systems, certain proteins in the corona may, on the other hand, facilitate processes such as adhesion and adsorption onto cell membranes, enhancing the biological activity of the nanodelivery system. Accordingly, specific and thorough investigations are needed to understand the direct correlation between the structure and the ensuing effects. To further complicate the issue, various proteins exhibit distinct binding affinities to NVs, dictating their sequence of adsorption onto the nanocarrier surface. Proteins with stronger affinities form the so-called hard corona, binding tightly to the surface compared to those forming the soft corona, which are endowed with lower affinities. Initially, the most abundant proteins adhere to the surface, replaced over time by higher-affinity proteins, a process termed the Vroman effect [18]. This phenomenon impacts NV biodistribution which, in turn, is crucial for in vivo applications. Among the most abundant components of almost every PC is human serum albumin (HSA). Due to its abundance in the bloodstream, HSA plays a crucial role in the pharmacokinetics of virtually all drugs, affecting their effectiveness, delivery rate, and toxicity [19]. Importantly, HSA emerges as a dominant protein that adheres to nanocarrier surfaces, a process typically viewed as benign or even advantageous, as it can not only reduce opsonization or inflammation but also prolong the circulation time of NVs, since the HSA corona can reduce the cellular uptake of NVs by macrophages [20,21,22,23].

In this perspective, understanding the interaction of HSA with our AD NMs is a crucial initial step for elucidating how these carriers are coated upon exposure to physiological environments, thus shedding further light on their fate, function, and potential biomedical applications. Accordingly, the formation of the HSA protein corona on the AD NMs was investigated through morphological, spectroscopic, calorimetric, and in silico techniques.

## 2. Materials and Methods

### 2.1. Reagents and Chemicals

Globulin- and fatty acid-free human serum albumin (product number A1887) was purchased from Sigma-Aldrich Inc. (Saint Louis, MO, USA). AD amphiphiles were synthesized and characterized as described in detail in our previous work [13].

### 2.2. Sample Preparation and Analytical Procedures

HSA stock solutions were prepared by dissolving a specific amount of protein in phosphate-buffered saline 1× (PBS, pH = 7.4). The protein concentration was determined via ultraviolet (UV) spectroscopy at a wavelength λ = 280 nm. AD nanocarrier stock solutions were prepared in PBS buffer at a final concentration of 2 mM.

### 2.3. Far-UV Circular Dichroism Spectroscopy

Far-ultraviolet circular dichroism (CD) spectra were recorded from 200 to 260 nm at a scan rate of 20 nm/min using a J-1500 spectropolarimeter (Jasco, Tokyo, Japan). The path length of the cell was 0.2 cm, with step size and bandwidth set to 0.5 and 1 nm, respectively. The resultant CD spectra represent the averaged data from three accumulations. The fractional composition of secondary structural elements within HSA, both in the presence and absence of AD, was determined using the CD Multivariate SEE program employing standard calibration models. The concentration of HSA was kept fixed at 2 µM, while AD concentration was varied in the range of 1–25 μM.

### 2.4. Steady-State and 3D Fluorescence Spectroscopy

All fluorescence spectroscopy (FS) measurements were carried out using a FP-8350 spectrofluorometer (Jasco, Tokyo, Japan) in a 1.0 cm quartz cell. The excitation wavelength (λ_exc_) was set to 280 nm, and the fluorescence spectra were recorded in the range of 300−450 nm at 298 K using 5 and 10 nm slit widths for the emission and the excitation bandwidth, respectively. During all fluorescence quenching titrations, the concentration of HSA was kept fixed at 2 µM, while AD concentration was varied in the range of 2–20 μM. Each dendrimer/protein solution was allowed to incubate for 2 h before recording the corresponding fluorescence spectrum. Additionally, 3D fluorescence spectroscopy was performed following the aforementioned protocol, with an excitation wavelength ranging from 220 to 350 nm and recording the spectrum in the range between 220 to 500 nm at 298 K.

### 2.5. Isothermal Titration Calorimetry Studies

Isothermal titration calorimetry experiments were performed with a MicroCal PEAQ-ITC calorimeter (Malvern Panalytical, Malvern, UK) at 298 K (cell volume = 208 μL). The thermodynamics associated with HSA/AD complex formation were investigated in PBS-buffered solutions. Specifically, a solution of HSA (30 μM, sample cell) was subjected to 19 step-by-step injections of 2 µL volume of AD (900 μM, syringe). In this way, it was ensured that AD was always found at a concentration above the CMC throughout the titration, as described in our previous works [24,25,26]. Prior to each experiment, the solutions and buffer were degassed for 30 min at room temperature under stirring at 700 rpm. Control experiments were performed to measure nonspecific heats, which were subsequently subtracted from the relevant dataset to obtain corrected integrated data. The experimental data acquired from the ITC experiment were analyzed using the “one set of sites” model. Subsequently, the fitted data were graphically depicted, with the *y*-axis denoting the heat generated in each injection and the *x*-axis representing the molar ratio between the concentration of albumin and the concentration of AD nanomicelles during titration within the sample cell. The concentration of AD nanomicelles was determined by employing the mass conservation principle, as outlined by the following equation [27,28]:

[AD_tot_] = [AD_mon_] + N_agg_ × [AD_mic_]. (1)

where [AD_tot_] is the total concentration of AD in the sample cell during the ITC experiment, [AD_mon_] is the concentration in its monomeric state, N_agg_ corresponds to the micellar aggregation number and [AD_mic_] is the concentration in its fully n-meric state. All experiments were conducted in triplicate.

### 2.6. Dynamic Light Scattering and Zeta Potential Measurements

All measurements of nanomicelle size and zeta (ζ)-potential were performed by dynamic light scattering (DLS) on a Zetasizer Nano-ZS (Malvern, UK) at 25 °C. AD NMs and HSA were dissolved in PBS and the measurements were performed using a 5 mV He-Ne laser (operating wavelength λ = 633 nm, scattering angle θ = 173°).

### 2.7. Transmission Electron Microscopy 

The morphology of the HAS/AD complex was examined using transmission electron microscopy (TEM). Briefly, a 5 mL volume of the complex solution was deposited onto a carbon grid and allowed to dry for 1 h. Subsequently, the grid was stained with uranyl acetate and imaged using a Philips EM 208 (Philips, Eindhoven, The Netherlands) electron microscope operating at 100 kV. The imaging system was equipped with a Quemesa camera from Olympus Soft Imaging Solutions (Hamburg, Germany) and utilized the RADIUS 2.3 software for image acquisition.

### 2.8. Molecular Simulations

To characterize both the isolated form and the self-assembled state of the AD amphiphile, a well-established procedure described in our prior work was adopted [13,29]. Accordingly, gaff2 atom types were assigned and partial charges were determined via the RESP method using the RED server [30,31,32]. The structure of HSA was obtained from the protein data bank (PDB, entry 4k2c) and parameterized with the AMBER ff14SB forcefield [33]. The Delphi 8.5 software was exploited to assess the distribution of electrostatic potential around the HSA surface, aiding in the appropriate orientation of the protein molecules to the charged nanomicelles. Parameters such as internal dielectric, scale, perfil, and ionic strength were set to the values of 4, 2.0, 70.0, and 0.15 M, respectively. By employing the same set of parameters and by setting the surfpot parameter to 4.0 Å, Delphi 8.5 was used for the calculation of the (ζ)-potential of the systems in the presence of explicit ions. The resulting AD/HSA molecular system was then solvated in a TIP3P water box extending at least 15 Å from each solute molecule [34]. Sodium and chlorine atoms were added to achieve a physiological concentration of 0.15 M. The complete protocol details can be found in our previous publications [13,29]. In brief, each system underwent energy minimization followed by gradual heating to 300 K at a pressure of 1 atm. Subsequently, a series of 5 simulations with gradually lowered positional restraints applied to the heavy atoms of the solute were performed in the isobaric–isothermal (NPT) ensemble. Unrestrained molecular dynamics (MD) simulations in the NPT ensemble were next conducted for 1 μs, with data collected from the last 100 ns of simulation where a stable complex was achieved. The energetic analysis was performed using the Molecular Mechanics/Poisson-Boltzmann Surface Area (MM/PBSA) approach implemented in the MMPBSA.py module of the AMBER22 suite, with an internal dielectric constant of 16 to accurately represent the highly charged environment at the binding interface [35,36,37]. Structural investigations were carried out using the AMBER22 main program CPPTRAJ and in-house developed Python scripts [38]. All simulations were executed with AMBER 22 running on our own GPU/CPU hybrid cluster and the pre-exascale Tier-0 EuroHPC Leonardo supercomputer (CINECA, Bologna, Italy) [37,39].

## 3. Results and Discussion

### 3.1. Experimental Binding Analysis of the HSA/AD Complex

#### 3.1.1. Fluorescence Spectra and Binding Constants of the HSA/AD Complexes

**Figure 2:**
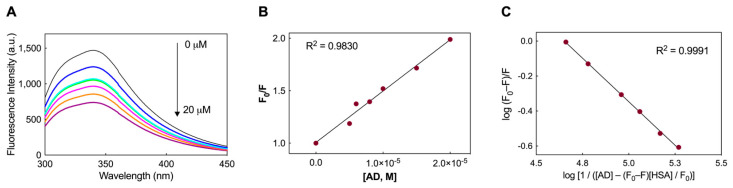
(**A**) Fluorescence emission spectra recorded for the HSA/AD NM systems. The spectra include measurements for free HSA (2 μM, black) and HSA at 2 μM with varying concentrations of AD NMs (5 μM in blue, 6 μM in cyan, 8 μM in magenta, 10 μM in orange, and 20 μM in purple). (**B**) Stern-Volmer plot illustrating the fluorescence quenching of HSA induced by the AD NMs (R^2^ = 0.9830). (**C**) Plots of log(F_0_ − F)/F vs. log[1/([AD)] − (F_0_ − F)[HSA]/(F_0_)]) for HSA fluorescence quenching by AD NMs (R^2^ = 0.9991).

Human serum albumin is composed of a single chain containing 585 amino acids, with a unique tryptophan residue (W214) located within subdomain II A. The emission from W214 primarily dictates the fluorescence spectra of HSA in the UV range. Upon interaction with other molecules, alterations in tryptophan fluorescence may arise, influenced either by conformational changes in the protein or direct quenching effects. Accordingly, changes in HSA fluorescence were monitored to investigate the binding of HSA with the AD nanomicelles, as shown in Figure 2A. The most notable change observed in the fluorescence spectrum upon addition of AD was the quenching in fluorescence intensity (Figure 2A). Increasing concentrations of AD in the experiment led to a linear decrease in tryptophan fluorescence. Therefore, the quenching behavior could be analyzed using the Stern–Volmer equation as follows:

F_0_/F = 1 + K_SV_ × [Q]. (2)

In the equation above, F_0_ represents the fluorescence intensity without the quencher, while F denotes the intensity with the quencher present. K_SV_ stands for the Stern–Volmer quenching constant, and [Q] indicates the concentration of the quencher. The equation assumes a linear relationship between F_0_/F and [Q] (see insert in Figure 2A), with the slope representing the value of K_SV_. In the present case, the calculated value of K_SV_ for AD is 4.9 (±0.2) × 10^4^ M^−1^.

Under the assumption that HSA and AD interact via an independent binding site within the protein structure, the binding constant (K_D_) values can be estimated from the plot in Figure 2B by using the following modified version of the Stern-Volmer equation [40]:

log(F_0_ − F)/F = log(1/K_D_) − log[1/([AD] − (F_0_ − F)[HSA]/F_0_)]. (3)

where F_0_ and F represent the fluorescence intensities as in the classic Stern-Volmer equation, [AD] denotes the increasing concentrations of the nanovector utilized in the experiment, while [HSA] is the fixed protein concentration of HSA (2 μM) adopted in the quenching assay. The K_D_ value was determined by assessing the linear correlation between the two logarithmic terms in the modified Stern-Volmer equation. This analysis resulted in an affinity value of 22.9 ± 4.2 μM, indicative of a moderate interaction between the nanomicelles and the serum protein [41,42].

#### 3.1.2. Isothermal Titration Calorimetry Binding Analysis

**Figure 3:**
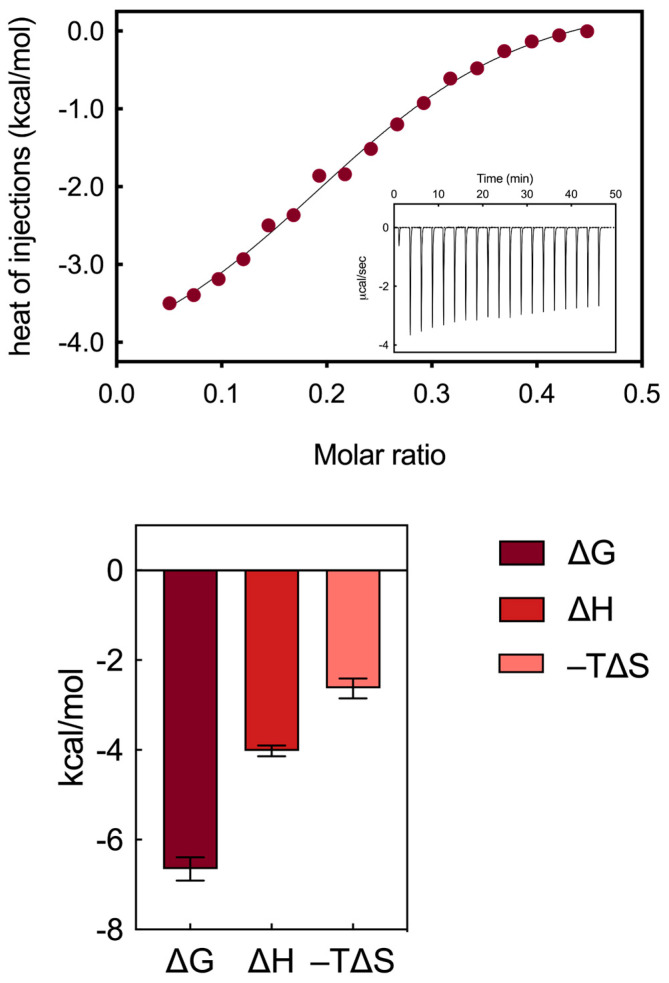
(**Upper panel**) ITC plot for the titration of HSA with AD nanomicelles (inset shows the measured heat power vs. time elapsed during HSA/AD binding). (**Bottom panel**) Summary of thermodynamic data extracted from ITC for the binding of AD nanosystems to HSA.

An isothermal titration calorimetry study was conducted to describe and quantify the interactions between the self-assembling AD nanocarrier and HSA in aqueous solutions at physiological pH (7.4). Various ITC conditions were explored to accurately determine the thermodynamic binding parameters between HSA and AD, aiming to unveil the interaction mechanisms between these two entities. This comprehensive analysis involved not only the assessment of binding affinity, expressed through the dissociation constant (K_b_) and the relevant Gibbs free energy (ΔG), but also the enthalpic (ΔH) and entropic (−TΔS) components, which characterize the interaction mechanism. Upon examination of the raw thermogram (Figure 3, upper panel insert), it is evident that each injection of AD, administered in discrete amounts, correlates with a negative heat flow peak, indicating an exothermic reaction between the protein and the nanocarrier. The determination of K_b_ values can be directly inferred from the sigmoidal profile of the integrated ITC data (Figure 3, upper panel), specifically from the slope at the inflection point. The binding strength obtained, represented by a mean affinity value of 13.4 ± 3.1 µM, indicates a moderate binding interaction between the serum macromolecule and the AD nanomicelles. Remarkably, the values obtained from the ITC experiments are quantitatively consistent with the results obtained from the fluorescence assay for the HSA/AD complexes.

The analysis of the complete binding thermodynamics (Figure 3, bottom panel) allowed for a detailed understanding of the interaction mechanism between the AD nanocarriers and HSA. Notably, the primary outcome of this analysis reveals the binding of the AD/HSA complex to be thermodynamically spontaneous, as indicated by the negative value of ΔG (−6.65 ± 0.26 kcal/mol, Figure 3). Subsequently, by integrating the obtained findings with the distinctive molecular determinants within the HSA/AD complex, the main interaction mechanisms between the AD nanovector and HSA could be delineated as follows:

1. robust electrostatic interactions are established between the positively charged terminal groups of the AD NMs and the side chains of some aspartic and glutamic acid residues within HSA;
2. a limited network of hydrogen bonds (HBs) forms between the internal groups of the AD molecules and specific amino acid residues of the serum protein, facilitating the exposure of side chains that serve as counterparts for the formation of proton bridges. Notably, within the AD dendron, the oxygens of the internal amide groups serve a hydrogen bond acceptor, with the -NH moieties of the same groups acting as HB donors;
3. these two types of interactions are mainly enthalpic in nature, as indicated by the favorable ΔH value obtained from the titrations (−4.02 ± 0.12 kcal/mol, Figure 3).

A couple of other not negligible interactions can be taken into account to complete the thermodynamics scenario. In particular, the following:

1. weak hydrophobic interactions occur between the hydrocarbon/aliphatic components of the nanocarrier heads and the side chain of hydrophobic amino acids of HSA;
2. secondary hydrophobic alterations occur due to bond formation at the respective surfaces, leading to the release of water molecules, ions and counterions. The latter two interactions contribute to favorable entropic changes, enhancing the stability of the interaction between the protein and the NMs, as demonstrated by the value of −TΔS = −2.63 ± 0.22 kcal/mol (Figure 3).

### 3.2. Structural and Thermal Perturbation of HSA upon AD Binding

#### 3.2.1. Determination of the HSA Secondary Structure and Melting Temperature through Circular Dichroism Spectroscopy

**Figure 4:**
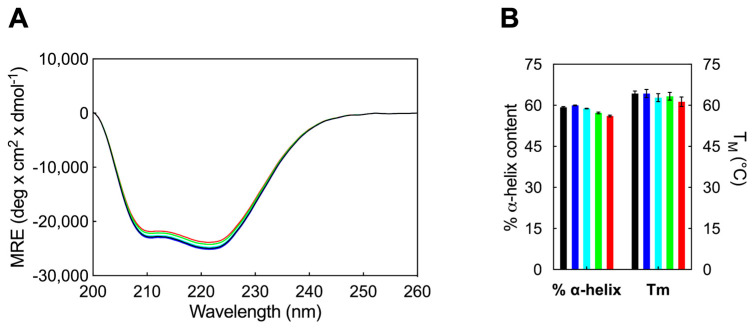
(**A**) Far-UV CD spectra of HSA alone (black line) and in the presence of AD in concentration of 1 µM (blue line), 5 µM (cyan line), 10 µM (green line) and 25 µM (red line). CD experiments were carried out in PBS buffer solution at 25 °C, with [HSA] maintained at 2 μM. (**B**) The α-helical content (left *y*-axis) and melting temperature (right *y*-axis) of HSA are depicted in the absence (black columns) and presence of AD at concentrations of 1 µM (blue columns), 5 µM (cyan columns), 10 µM (green columns), and 25 µM (red columns).

Circular dichroism spectroscopy is a valuable technique for elucidating protein structures and examining conformational changes induced by ligand interactions [43]. In the far-UV CD spectra of HSA, two negative bands are observed at 208 (π-π*) and 220 nm (n-π*), indicative of an α-helix-rich protein secondary structure. To ascertain the impact of AD nanovector binding on the secondary structure of HSA, CD spectra of free HSA and its AD complexes (Figure 4A) were recorded. These experiments were conducted to compare the percentage of α-helix content in free HSA and in the presence of the nanovector, at concentrations below (monomer form) or above (nanomicelles) its critical micelle concentration (CMC), with CD spectra values recorded between 200 and 260 nm at 25 °C. The percentage α-helicity of HSA was estimated from the Mean Residue Ellipticity (MRE) values at 208 nm using the following equation:

MRE_208_ = (observed CD (mdeg))⁄(10C_p_nl) (4)

α−helix (%) = [(−MRE_208_ − 4000)/(33000 − 4000)] × 100. (5)

in which MRE is the value of the mean residue ellipticity at λ = 208 nm (expressed in deg cm^2^ dmol^−1^), C_p_ is the molar concentration of HSA (2 μM), n denotes the number of amino acids in the protein primary sequence (585 for HSA), l is the path length of the CD cell (0.1 cm), while 33,000 and 4000 (in deg cm^2^ dmol^−1^) correspond to the MRE_208_ values of pure α-helical and β-sheet/random coil structures, respectively. The findings of this analysis are depicted in Figure 4B. The CD spectra yielded a percentage of α-helix of 59.3 for the unbound HSA, aligning with previous studies reported in the literature [44].

The results of the CD experiments indicate that the secondary structure of HSA remains unaffected by the interaction with AD at concentrations below its CMC (e.g., 1 µM), revealing that AD in its monomeric form does not induce significant conformational changes in the secondary structural motifs of the protein. Upon increasing AD concentration above its CMC, a slight decreasing trend is observed, resulting in a final α-helix percentage of 56.1% (Figure 4A), measured at the maximum AD concentration of 25 µM.

Furthermore, temperature-dependent CD spectroscopy was employed to investigate the potential impact of AD on altering the melting temperature (T_M_) of HSA. T_M_ values were determined by recording CD spectra at 222 nm across a temperature range of 25 °C to 95 °C, with the addition of nanocarriers below and above their CMC. The obtained results are presented in Figure 4B. Consequently, the assessment of HSA thermal stability, based on T_M_ estimation, yielded consistent findings with the standard CD spectra. In fact, there were no significant fluctuations observed in the T_M_ value of HSA in the presence of AD monomers or at lower molar ratios of HSA/AD nanomicelles (Figure 4B). However, at higher concentrations, AD NMs were capable of reducing the albumin T_M_ by approximately 3 °C. Thus, it is only in the presence of a large quantity of AD amphiphiles in their self-assembled form that HSA exhibits a slight decrease in thermal stability, aligning with the modest reduction observed in α-helical content (Figure 4B).

In summary, the interaction between serum albumin and AD nanomicelles did not induce any substantial structural alterations within the protein structure. This observation is consistent with the moderate binding strength of the HSA/AD complexes, as elucidated previously through fluorescence spectroscopy and ITC analysis.

#### 3.2.2. Three-Dimensional Fluorescence Spectroscopy

**Figure 5:**
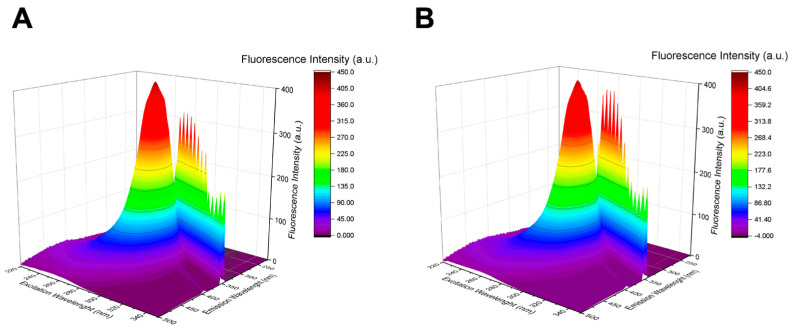
3D fluorescence spectra of HSA in absence (**A**) and in presence (**B**) of AD nanomicelles (10 µM).

3D fluorescence spectroscopy serves as a further, valuable tool for probing changes in HSA conformation. The fluorescence intensity of the primary peak in the 3D FS spectrum is associated with the polarity of the microenvironment and the secondary structure of the protein. Notably, the peak (λ_ex_/λ_em_ = 280/343 nm/nm) predominantly reflects the spectral characteristics of W214 of HSA, while the other peaks correspond to the first-order Rayleigh scattering peak (λ_ex_ = λ_em_) [19]. To corroborate the findings obtained from CD spectroscopy, 3D FS spectra (Figure 5) were recorded.

As seen in Figure 5, no significant shifts in the peaks of HSA are observed in the analysis of the 3D FS spectra in the presence of AD nanomicelles. These results suggest that there are no notable alterations in the structure of HSA around the tryptophan residue located at position 214 following interaction with AD. Consequently, it can be inferred that the binding between HSA and the NVs likely occurs predominantly at the external surface of the proteins, particularly with the charged surface of the nanomicelles.

#### 3.2.3. Transmission Electron Microscopy and Dynamic Light Scattering Analysis

**Figure 6:**
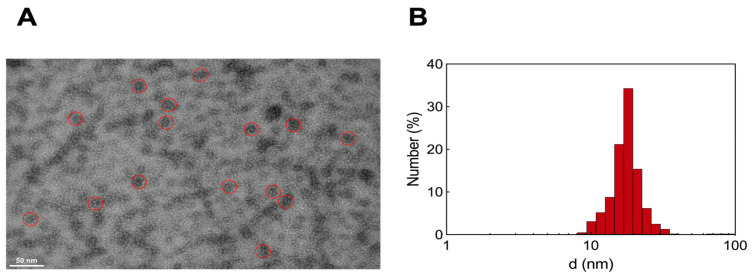
(**A**) TEM images of HSA/AD complexes. Red circles highlight three representative HSA/AD NM structures in each TEM ensemble. (**B**) Hydrodynamic diameter (d) of HSA/AD complexes at 25 °C as measured by DLS.

Transmission electron microscopy (TEM) analysis of the HSA/AD NM systems revealed the formation of a molecular complex exhibiting a diameter approximately 20 nm with a spherical morphology (Figure 6A). Dynamic light scattering (DLS) experiments conducted in aqueous solution provided measurements of the hydrodynamic diameter (d) and the ζ-potential (ζ), yielding values of 17.6 ± 0.3 nm (Figure 6B) and +8.3 mV, respectively. In our previous work [13], the ζ-potential for pristine AD NMs was recorded as +27 mV, while literature data suggests that pure HSA typically exhibits negative ζ values around −15 mV [45]. This suggests that although the positively charged AD NMs primarily interacts with the negative residues of HSA, each HSA molecule within the complex aligns in a manner where its more positively charged areas are exposed to the surrounding solvent. This evidence, along with the calculated N_agg_ for the AD NMs [13] and the molar ratio detected from the ITC experiments, supports a plausible composition of six serum proteins around a single AD nanoassembly, consistent with experimental results obtained in similar systems [46].

### 3.3. Computational Studies

#### 3.3.1. Binding Analysis of the HSA/AD Complex

**Figure 7:**
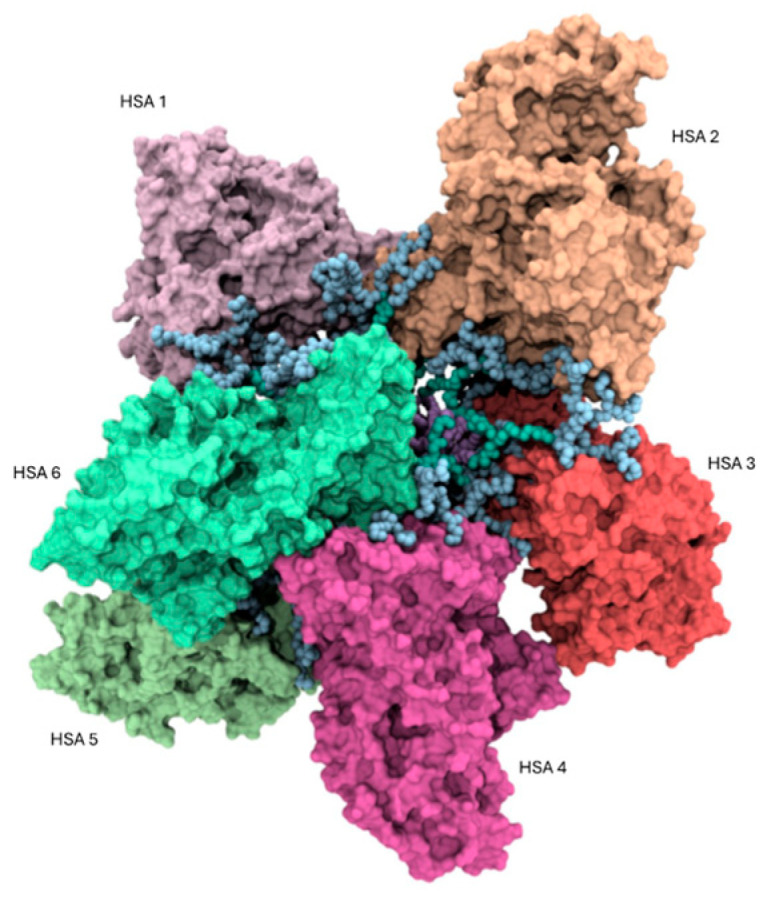
Atomistic representation of the HSA/AD (6/1) complex. Each protein structure is depicted as a colored surface, while the atoms of the AD nanomicelle are represented as spheres colored according to the scheme adopted in Figure 1.

Based on the experimental findings, it is likely that HSA and the AD NMs primarily interact through surface interactions involving the polar external amino acid residues of the protein and the hydrophilic, positively charged heads of the NVs. To elucidate the interactions between these two molecular entities at the atomistic level, an investigation was initiated by examining the electrostatic potential surrounding the serum protein using Delphi 8.5 [47] in conjunction with UCSF Chimera 1.17.3 [48]. While the serum protein typically exhibits an abundance of negatively charged amino acids under physiological conditions, the analysis revealed that these residues are not uniformly distributed on its surface. Nonetheless, a distinct surface with a notable overall negative potential was identified, particularly over the IA, IIA, and IIB domains of HSA (Figure S1), thus indicating a preferential site for binding with the positively charged AD nanomicelle. This facilitated the formation of the initial complex between HSA molecules and the AD nanomicelle. Consistent with our experimental evidence and literature data on a similar system [46], the complex is composed of six protein molecules making up the PC around one self-assembled AD NM. The determination of the surface electrostatic potential enabled us to validate the 6:1 stoichiometry of the HSA/AD complex. Specifically, the ratio between the solvent accessible surface area (SASA) of the AD nanomicelle (35,618 Å^2^) and the SASA corresponding to the region of HSA exhibiting the most negative potential distribution (Figure S1, 5835 Å^2^) within a single protein yielded a value of 6.1, closely resembling the experimentally determined HSA/AD ratio. Consequently, after establishing the appropriate orientation of HSA molecules relative to AD nanomicelle, six HSA molecules were manually positioned in proximity to the AD nanomicelle. Each HSA molecule was placed at a minimum distance of 10 Å from the AD nanomicelle, with the protein molecules evenly distributed on the surface of the AD nanomicelle (Figure 7).

**Figure 8:**
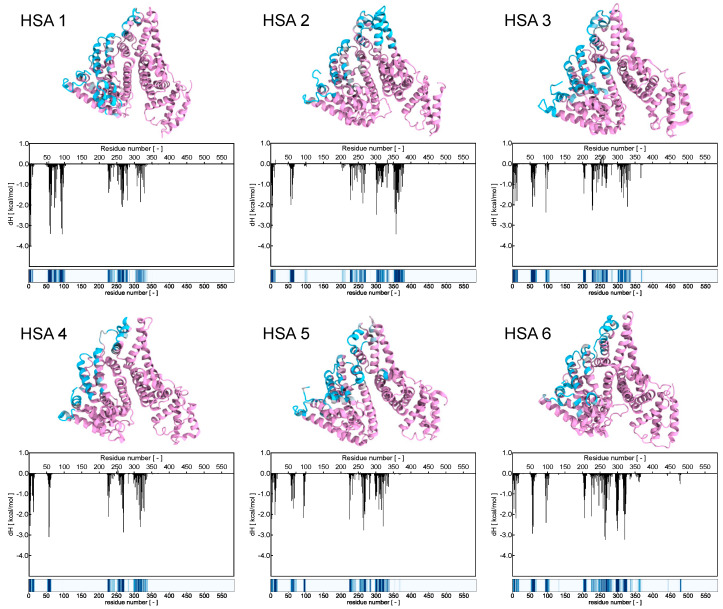
The six panels represent each HSA bound to the AD micelle in Figure 7. (**top panels**) HSA protein is represented by its secondary structure, and the aminoacid residues are colored in pink (no contact) or cyan (consistent contacts). (**middle panels**) the enthalpic free energy decomposition for each HSA residue as obtained from the MM/PBSA analysis. (**bottom panels**) The interaction fingerprint is based on the frequency of interaction of each HSA residue with the AD micelle. Fingerprints are colored based on the contact frequency with shades of blue: white representing 0 contact frequency while dark blue representing a constant contact throughout the MD trajectory.

Focusing on the primary interactions at the atomic level between HSA and the AD NM, the initial phase involved assessing the contacts between the micelle’s atoms and the residues of the protein along the corresponding equilibrated MD trajectory. Essentially, this process entailed determining the closest distance between every amino acid on the HSA molecules and any atom within the micelle for each snapshot of the MD simulation (Figure S2). Initial observations unveiled a consistent interaction pattern, where the HSAs making up the protein corona appeared to contact the NM at very similar regions and amino acids. Subsequently, the frequency of protein nm contacts was calculated by defining a contact as a scenario where the minimum distance between two interacting atoms (belonging to the protein and the NM, respectively), was less than 6.5 Å in any given MD frame. This approach resulted in the generation of a more detailed fingerprint of the interactions between each protein and the AD NM, as illustrated in Figure 8.

Examination of these binding fingerprints revealed consistent interaction patterns across all HSA molecules (Figure 8, bottom panels). To quantitatively characterize these patterns in terms of energy, a MM/PBSA analysis was conducted. This analysis allowed for the deconvolution of the enthalpic binding energy into individual contributions from each amino acid within the protein. Subsequently, comparison between the interaction fingerprints (Figure 8, bottom panels) and the energy contributions of each amino acid (Figure 8, middle panels and Table S1) revealed a significant correlation between the energy profiles and the structural data.

For a deeper understanding of the interaction between HSA and the AD NV, the number of charged AD groups involved in stable salt bridges (SBs) with the protein’s residues. These SBs were deemed significant if they persisted for at least 50% of the MD simulation duration. Results revealed that approximately 72.8% of the charged terminals on the AD NM formed stable SBs with negatively charged (i.e., aspartic and glutamic acid) residues on HSA. While salt bridges play a pivotal role in binding and represent a significant portion of the interactions identified through contact and energy analysis, they are not the sole contributors. Hydrogen bonds and van der Waals forces also contribute to the stabilization of the macromolecular complexes as represented in Figure S3. Although the interaction sites are distributed widely across the protein’s surface (Figure 8, upper panels), two regions of HSA appear particularly crucial for binding with the positively charged branches of the AD NM. These regions include the helix containing residues E252, D255, D256, D259, E266, and D269, as well as the small loop containing residues E60 and D63. These regions form highly stable salt bridges with the nanomicelle terminal nitrogen in each HSA molecule comprising the protein corona (Figure 8).

#### 3.3.2. Binding Analysis of the HSA/AD Complex

**Figure 9:**
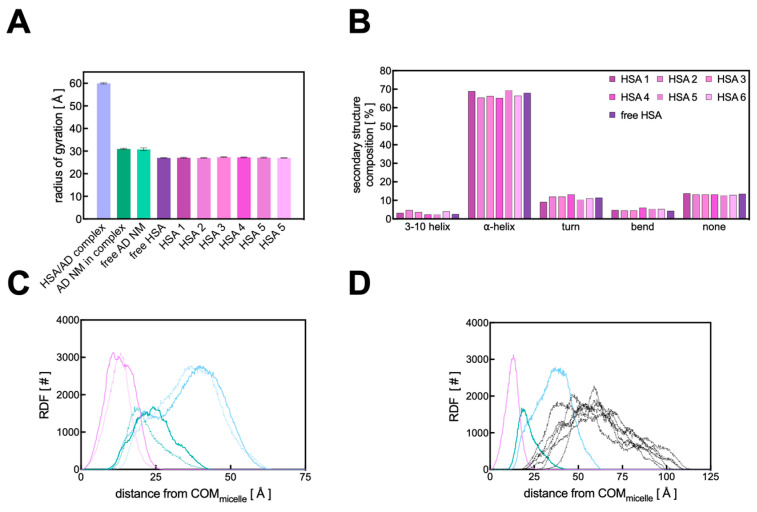
(**A**) Comparison of the radius of gyration (R_g_) values for the HSA/AD complex, the AD NM within the complex, a free AD NM, a free HSA molecule, and each of the 6 HSA chains within the HSA/AD assembly. (**B**) Comparison of the secondary structure composition for the 6 HSA molecules in the AD complex and an isolated HSA chain. (**C**) Radial distribution functions of the AD NM atoms (tail in purple, linker in aquamarine, head in cyan), showing variations between unbound and complexed states. Dotted lines represent the RDF values for the unbound AD NM, while the solid lines represent the values for the AD NM in complex with HSA. (**D**) RDF of the complete HSA/AD complex relative to the center of mass of the AD NM. The RDF of the protein atoms are represented as black lines.

Further analysis of the atomistic MD data for the HSA/AD NM complex revealed that the overall sizes of both the HSA chains and the AD NM remain unchanged upon the formation of the protein corona, as evidenced by the corresponding value of the radius of gyrations (R_g_). This parameter serves as an indicator of the compactness of a given molecular (super)structure and directly reflects its size in solution. As illustrated in Figure 9A, there is virtually no alteration in the micelle’s R_g_ upon formation of the HSA PC, with R_g_ values of 30.82 ± 0.62 Å for the AD NM alone and 30.98 ± 0.32 Å within the HSA complex.

As previously documented by SAXS experiments [13], the AD NMs exhibit a triaxial ellipsoid shape in solution. Accordingly, the analysis of the gyration tensor’s principal components performed in the present work provided further insights into the overall geometry of the HSA/AD complex. The square roots of the gyration tensor’s eigenvalues represent the characteristic semi-axis lengths of the ellipsoid characterizing both the AD NM and its complex with HSA. Remarkably, upon complexation, the AD micelle maintains its triaxial ellipsoid configuration (with semi-axis lengths of 45.8 ± 0.4 Å, 39.78 ± 0.3 Å, and 37.18 ± 0.3 Å), indicating that its interaction with serum proteins does not induce structural alterations in the AD nanostructure, despite protein distribution across its surface. Notably, when examining the overall 6:1 HSA/AD complex, its shape resembles that of an oblate spheroid, featuring a discernible minor axis (with semi-axis lengths of 81.8 ± 0.5 Å, 80.2 ± 0.3 Å, and 71.4 ± 0.7 Å).

From the perspective of the protein, its interaction with the micelles does not seem to notably perturb its overall structure. As demonstrated in Figure 9A, the size of each protein when bound to the AD micelle (as indicated by the R_g_ value) remains largely unchanged upon complex formation. In fact, the calculated R_g_ values for different HSA chains range from 26.98 to 27.35 Å, with a maximum standard deviation of 0.18 Å.

These values align with the R_g_ value computed for a single HSA under similar conditions (27.01 ± 0.16 Å) and are consistent with previously reported literature data on HSA [49]. To delve deeper into the potential impacts of AD nanomicelle binding on the internal structure of HSA, the secondary structure content of the proteins, both free and bound to the AD NM, was analyzed using the DSSP algorithm [50], which assigns secondary structures to amino acids based on their 3D coordinates. The secondary structure of a single HSA simulated under similar conditions predominantly comprises α-helices (68.0%), along with some turns (11.5%), and no β-sheets, consistent with existing literature [44]. In complex with the AD NM, the α-helix percentages vary between 65.3 and 69.4%, and the turns between 9.2 and 13.2%, with no β-sheet formation observed. These findings suggest that the interaction between the HSA chains and the AD NV does not significantly alter the arrangement of protein secondary structure, as evidenced in Figure 9B. Furthermore, considering again the 6:1 full HSA/AD NM complex, the relevant computed R_g_ is 60.01 ± 0.27 Å (Figure 9A). This value implies that the proteins and the AD NM do not interact as rigid objects, for which a higher R_g_ value would be expected.

The surface-averaged electrostatic potential (SAEP) was next calculated to estimate the ζ-potential potential of the HSA/AD NM complex. Initially, the ζ-potential of the pure HSA protein was determined to be −11.5 mV, while the value for the AD NM alone was calculated to be 30.48 mV. Upon complexation with HSA, the overall positive ζ-potential of the complex decreased to 10.2 mV. This suggests that although the positively charged AD NM could be largely neutralized by the negative residues of HSA, each HSA molecule in the complex is oriented with its more positively charged regions (Figure S1, right panel) facing the solvent. This unique orientation of HSA thus contributes to the observed positive ζ-potential value of the supramolecular assembly.

Finally, to complete the investigation of the structure of the HSA/AD complex and the relevant interactions, the radial distribution function (RDF) analysis was performed. This method involves selecting a reference point, typically the center of mass (COM) of a large molecule or a specific residue, and then calculating the distribution of atoms from another molecular entity as the distance from this reference point increases. Initially, this technique was employed to examine the arrangement of each AD amphiphile within the nanomicelle itself and to determine whether the adsorption of the HSA proteins onto the NV altered its internal organization (see Figure 1 for details). As shown in Figure 9C, the internal distribution of the AD NM atoms remains largely unchanged upon complexation with HSA, consistent with the previously described results. By plotting the RDF of HSA atoms around the COM of the AD nanomicelle (Figure 9D), it becomes evident that there is a high degree of interpenetration between the proteins and the NV, which supports the previously described R_g_ value estimated for the complex. Furthermore, upon observing the distribution of HSA atoms relative to the different components of the NV, a strong interpenetration of the proteins within the polar heads of the AD nanomicelle is observed, aligning with the overall binding mechanism underlying the HSA/AD complex.

## 4. Conclusions

Dendrimers, which are complex compounds with highly branched nanostructures, offer precise control over their size, shape, and functionality, making them unique macromolecules. Their uniform architecture provides several advantages, such as high surface-to-volume ratios and monodispersity, which are crucial for their applications in drug delivery and diagnostics. However, challenges arise due to structural anomalies during dendrimer synthesis, which can affect their clinical translation. Recent research efforts have focused on self-assembly strategies to address these limitations. One promising approach involves the use of amphiphilic dendrimers, which form stable nanomicelles suitable for drug delivery. However, understanding how these dendrimers interact with biological environments, particularly with human serum albumin, is essential for realizing their biomedical potential.

Accordingly, in the present effort the interaction between HSA and the recently designed self-assembling, amphiphilic dendrimer AD was investigated using various experimental techniques. Fluorescence spectroscopy revealed a linear decrease in tryptophan fluorescence of HSA upon binding to AD nanomicelles, indicating quenching behavior, which was analyzed using the Stern–Volmer equation. The calculated Stern–Volmer quenching constant (K_SV_) for the AD NMs was determined to be 4.9 × 10^4^ M^−1^, while the affinity value obtained from the modified Stern–Volmer equation was equal to 22.9 ± 4.2 μM, indicating a moderate affinity between the protein and the nanovector. Additionally, isothermal titration calorimetry confirmed the moderate entity of the interaction between HSA and the AD NMs, with a mean affinity value of 13.4 ± 3.1 µM. Thermodynamic analysis indicated thermodynamically spontaneous binding with favorable enthalpic contributions primarily from electrostatic interactions and hydrogen bonding. Circular dichroism spectroscopy showed no significant structural changes in HSA upon interaction with AD below its critical micellar concentration, with only slight decreases in α-helical content and thermal stability observed at higher AD concentrations. Three-dimensional fluorescence spectroscopy further supported these findings, indicating no significant alterations in HSA structure around the HSA tryptophan residue W214 upon AD binding. Computational analysis of the HSA/AD complex binding revealed surface interactions between polar amino acid residues of HSA and the hydrophilic, positively charged heads of the nanovector. Delving into atomistic details, electrostatic potential around HSA was analyzed, revealing a predominantly negative potential over specific domains, ideal for binding with AD nanomicelles. Moreover, molecular dynamics simulations unveiled consistent interaction patterns between HSA and the AD NV at the atomistic level. Further energetic analysis elucidated enthalpic contributions, with stable salt bridges playing a pivotal role. Finally, structural analysis indicated minimal alteration in the overall structure of HSA upon complexation with AD micelles, corroborated by radial distribution functions and zeta potential measurements. Overall, this study sheds light on the binding mechanisms and thermodynamic parameters governing the interaction between HSA and the self-assembled AD nanovector, offering valuable insights into their potential biomedical applications.

In a broader context, the observed moderate binding pattern between HSA and the AD nanomicelles holds considerable implications for pharmaceutical research, especially within the realm of drug delivery. Understanding the nature of the interactions between these entities is paramount for refining drug delivery systems and enhancing their efficacy. In the domain of drug delivery, a moderate binding pattern can be construed as advantageous, as it fosters three primary beneficial outcomes [19,51]:

(i) mitigated carrier-induced toxicity, since an excessive binding affinity between any drug vector and HSA can perturb normal protein functions. A moderate binding pattern serves to alleviate such risks, thus increasing the safety of the drug delivery system;
(ii) enhanced circulation stability, since moderate binding ensures a stable interaction between the drug carrier and HAS, and this, in turn, can enhance the circulation time of the drug delivery system in the bloodstream. This prolonged circulation time improves drug bioavailability and increases the likelihood of reaching target tissues;
(iii) diminished immunogenicity since nanovector binding to HSA may mitigate the recognition of the drug delivery system as foreign by the immune system. This effect could potentially decrease immune responses, such as clearance by the reticuloendothelial system or the initiation of inflammatory reactions.

Finally, understanding the binding mechanisms and thermodynamic parameters empowers researchers to engineer nanocarriers with customized properties, optimizing drug loading, circulation stability, and targeted drug release, thereby advancing therapeutic outcomes in pharmaceutical applications [52].

## Data Availability

The original contributions presented in the study are included in the article/Supplementary Materials, further inquiries can be directed to the corresponding authors.

## Supplementary Materials

The following supporting information can be downloaded at: https://www.mdpi.com/article/10.3390/pharmaceutics16040533/s1, Figure S1: Solvent-accessible surface of HSA; Figure S2: Average minimum distances.; Figure S3: Representative interactions between AD and HSA. Table S1: enthalpic free energy decomposition data; HSA_AD_complex.pdb: atomistic coordinates of the HSA/AD complex.
