# Supplementary material for: Molecular Ballet: Investigating the Complex Interaction between Self-Assembling Dendrimers and Human Serum Albumin via Computational and Experimental Methods
Source: Pharmaceutics. 2024 Apr 12;16(4):533. doi: 10.3390/pharmaceutics16040533 (PMC11054399; doi:10.3390/pharmaceutics16040533)
Supplement: Supplementary file 1 [file pharmaceutics-16-00533-s001.zip › pharmaceutics-2948229-supplementary.pdf]

Molecular Ballet: Investigating the Complex Interaction Between Self-Assembling Dendrimers and Human Serum Albumin via Computational and Experimental Methods

Gabriele Cavalieri [1†], Domenico Marson [1†], Nicoletta Giurgevich[1], Rachele Valeri[1], Fulvia Felluga[2], Erik Laurini [1,‡*] and Sabrina Pricl [1,3‡]

[1] Molecular Biology and Nanotechnology Laboratory (MolBNL@UniTS), DEA, University of Trieste, Piazzale Europa 1, 34127 Trieste, Italy

[2] Department of Chemical and Pharmaceutical Sciences, DSCF, University of Trieste, Via Giorgieri 1, Trieste 34127, Italy

[3] Department of General Biophysics, Faculty of Biology and Environmental Protection, University of Lodz, ul. Pomorska 141/143, 90-236 Łódź, Poland

* Correspondence: erik.laurini@dia.units.it;

† These authors equally contributed to the manuscript

‡ Senior co-authors

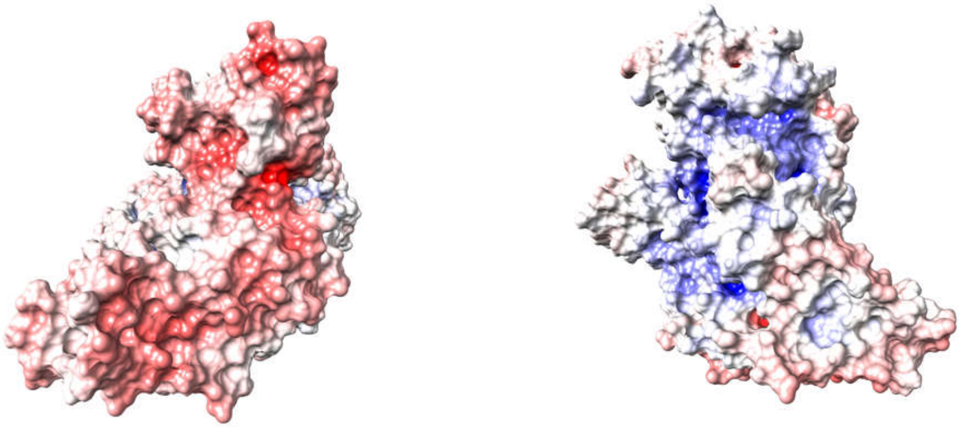

Figure S1. Solvent-accessible surface of HSA. HSA molecule is represented by its solvent-accessible surface, colored by the electrostatic potential. Areas predominantly negatively charged and positively charged (left and right panels, respectively) are shown.

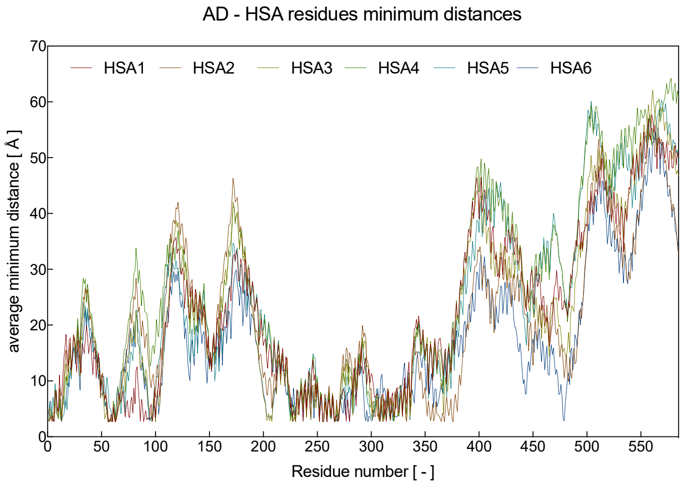

Figure S2. Average minimum distances. The average minimum distance between each aminoacid of each HSA and the micelle atoms.

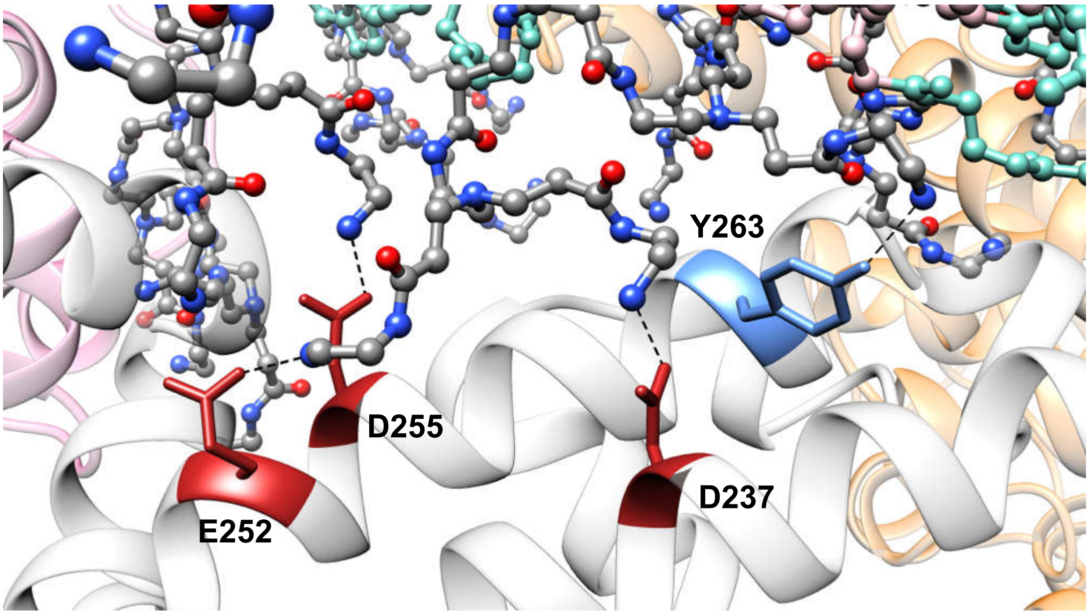

Figure S3. Representative interactions between AD and HSA. Salt bridges (SB) and H-bond (HB) are represented by black dotted line. The proteins are shown by colored ribbons while the AD NM is depicted in ball&stick style. The protein residues involved in SB or in HB are labelled and colored in firebrick or in cornflower blue, respectively. The AD head atoms are colored by element while the AD atoms belonging to linker and tail are colored according to the color scheme in Figure 1 of the main text.

Table S1. The enthalpic free energy decomposition values, obtained from the MM/PBSA analysis, for each HSA residue. All data are expressed in kcal/mol.

RESIDUE NUMBER	HSA 1	HSA 2	HSA 3	HSA 4	HSA 5	HSA 6
1	-0.66	-0.85	-1.04	-1.57	-1.16	-0.51
2	-2.24	-0.95	-0.39	-1.89	-0.29	-0.31
3	-2.47	-3.14	-1.22	-2.56	-2.23	-0.36
4	-4.00	-2.12	-2.05	-1.33	-1.40	-1.74
5	-1.22	-1.54	-0.93	-0.61	-0.12	-0.82
6	0.04	-0.07	-0.44	-0.14	-0.45	-0.79
7	-0.11	-0.16	-0.13	-0.06	-0.05	-0.08
8	-0.10	-0.04	-0.53	-0.12	-0.06	-0.10
9	-0.63	-0.35	-1.19	-1.09	-0.43	-1.92
10	-0.52	-0.66	-1.83	-1.09	-0.80	-2.19
11	-0.04	-0.02	-0.10	-0.13	-0.22	-0.14
12	-0.14	-0.04	-0.93	-1.33	-2.03	-0.35
13	-0.09	0.20	-1.81	-1.88	-1.39	-0.52
14	-0.03	-0.06	-0.17	-0.26	-0.84	-0.12
15	-0.01	-0.01	-0.03	-0.20	-0.72	-0.09
16	-0.01	0.00	-0.02	-0.04	-0.25	-0.24
17	-0.01	0.00	-0.01	-0.01	-0.02	-0.47
18	-0.01	0.00	-0.02	-0.02	-0.28	-0.06
19	-0.02	-0.01	-0.03	-0.03	-0.05	-0.04
20	-0.01	0.00	-0.01	-0.01	-0.01	-0.06
21	0.00	0.00	0.00	0.00	-0.01	-0.01
22	-0.01	-0.01	-0.01	-0.01	-0.02	-0.02
23	-0.01	0.00	-0.01	0.00	-0.01	-0.01
24	0.00	0.00	0.00	0.00	0.00	-0.01
25	-0.01	0.00	0.00	0.00	0.00	-0.01
26	-0.01	0.00	-0.01	0.00	0.00	-0.01
27	-0.02	0.00	-0.01	0.00	-0.01	-0.01
28	0.00	0.00	0.00	0.00	0.00	0.00
29	-0.01	0.00	-0.01	0.00	0.00	-0.01
30	-0.02	-0.01	-0.02	0.00	0.00	-0.03
31	-0.01	0.00	0.00	0.00	0.00	0.00
32	0.00	0.00	0.00	0.00	0.00	0.00
33	0.00	0.00	0.00	0.00	0.00	0.00
34	0.00	0.00	0.00	0.00	0.00	0.00
35	0.00	0.00	0.00	0.00	0.00	0.00
36	0.00	0.00	0.00	0.00	0.00	0.00
37	0.00	0.00	0.00	0.00	0.00	0.00
38	0.00	0.00	0.00	0.00	0.00	0.00
39	-0.01	0.00	0.00	0.00	0.00	0.00
40	0.00	0.00	0.00	0.00	0.00	0.00
41	-0.01	0.00	0.00	0.00	0.00	0.00
42	-0.02	0.00	0.00	0.00	0.00	0.00
43	-0.01	0.00	0.00	0.00	0.00	-0.01
44	-0.01	0.00	0.00	0.00	0.00	-0.01
45	-0.03	0.00	-0.01	0.00	-0.01	-0.01

46	-0.02	-0.01	-0.01	-0.01	-0.01	-0.01
47	-0.01	0.00	-0.01	-0.01	-0.01	-0.02
48	-0.04	-0.01	-0.01	-0.01	-0.01	-0.01
49	-0.10	-0.07	-0.07	-0.02	-0.04	-0.04
50	-0.02	-0.01	-0.04	-0.02	-0.01	-0.02
51	-0.03	-0.01	-0.06	-0.05	-0.02	-0.09
52	-0.07	-0.03	-0.09	-0.05	-0.01	-0.04
53	-0.14	-0.12	-0.78	-0.13	-0.04	-0.18
54	-0.12	-0.04	-0.90	-0.32	-0.07	-0.19
55	-0.15	-0.05	-0.67	-0.52	-0.01	-0.35
56	0.14	-0.19	-0.43	-0.39	-0.04	-1.06
57	-1.38	-1.58	-2.09	-3.09	-0.13	-2.94
58	-3.00	-2.01	-1.55	-2.62	-0.59	-2.52
59	-1.24	-0.25	-1.16	-0.65	-0.43	-0.60
60	-3.40	-0.23	-0.50	-0.71	-1.34	-2.60
61	-0.50	-0.18	-0.39	-0.09	-0.26	-0.30
62	-0.97	-1.02	-0.73	-0.30	-0.30	-0.38
63	-1.51	-1.71	-1.53	-0.23	-1.26	-0.20
64	-2.05	-0.70	-1.60	-0.05	-1.72	-0.16
65	-0.32	-0.56	-0.60	-0.03	-0.48	-0.05
66	-0.10	-0.15	-0.11	-0.05	-0.07	-0.08
67	-0.17	-0.14	-0.47	-0.02	-0.12	-0.19
68	-0.32	-0.03	-0.20	-0.01	-1.21	-0.04
69	-0.16	-0.02	-0.06	-0.01	-0.15	-0.02
70	-0.06	-0.01	-0.03	-0.01	-0.03	-0.02
71	-0.14	0.00	-0.01	0.00	-0.03	-0.01
72	-2.12	0.00	-0.04	0.00	0.08	-0.01
73	-0.23	0.00	-0.01	0.00	-0.02	-0.01
74	-0.11	0.00	-0.01	0.00	-0.01	-0.01
75	-1.36	0.00	-0.01	0.00	-0.02	-0.01
76	-1.66	0.00	0.00	0.00	-0.01	0.00
77	-0.10	0.00	0.00	0.00	0.00	0.00
78	-0.10	0.00	0.00	0.00	0.00	0.00
79	-0.74	0.00	0.00	0.00	0.00	0.00
80	-0.04	0.00	0.00	0.00	0.00	0.00
81	-0.26	0.00	0.00	0.00	0.00	0.00
82	-0.01	0.00	0.00	0.00	0.00	0.00
83	-0.01	0.00	0.00	0.00	0.00	0.00
84	-0.04	0.00	0.00	0.00	0.00	0.00
85	-0.07	0.00	0.00	0.00	0.00	0.00
86	-0.04	0.00	-0.01	0.00	0.00	-0.01
87	-0.14	0.00	-0.01	0.00	0.00	-0.01
88	-0.25	0.00	0.00	0.00	0.00	0.00
89	-0.93	0.00	-0.01	0.00	-0.01	-0.01
90	-1.82	0.00	-0.02	0.00	-0.01	-0.03
91	-0.71	0.00	-0.01	0.00	-0.02	-0.01
92	-3.14	0.00	-0.01	0.00	-0.02	-0.02
93	-3.12	0.00	-0.09	0.00	-0.12	-0.24
94	-3.42	-0.01	-0.53	-0.01	-2.17	-1.10
95	-0.52	-0.02	-0.70	-0.01	-1.07	-0.54

96	-0.41	-0.11	-2.38	-0.01	-1.59	-2.41
97	-0.94	-0.02	-0.01	0.00	-0.04	-0.72
98	-1.12	-0.01	-0.13	0.00	-0.41	-0.13
99	-0.07	-0.09	-0.28	0.00	-0.04	-0.27
100	-0.03	-0.06	-1.23	0.00	-0.05	-1.31
101	-0.17	-0.01	-0.17	0.00	-0.01	-0.14
102	-0.08	-0.01	-0.06	0.00	-0.02	-0.04
103	-0.02	-0.14	-0.39	0.00	-0.01	-0.57
104	-0.09	-0.01	-0.29	0.00	-0.01	-0.25
105	-0.02	0.00	-0.02	0.00	0.00	-0.02
106	-0.01	-0.01	-0.02	0.00	0.00	-0.02
107	0.00	0.00	-0.01	0.00	0.00	0.00
108	0.00	0.00	0.00	0.00	0.00	0.00
109	0.00	0.00	0.00	0.00	0.00	0.00
110	0.00	0.00	0.00	0.00	0.00	0.00
111	0.00	0.00	0.00	0.00	0.00	0.00
112	0.00	0.00	0.00	0.00	0.00	0.00
113	0.00	0.00	0.00	0.00	0.00	0.00
114	0.00	0.00	0.00	0.00	0.00	0.00
115	0.00	0.00	0.00	0.00	0.00	0.00
116	0.00	0.00	0.00	0.00	0.00	0.00
117	0.00	0.00	0.00	0.00	0.00	0.00
118	0.00	0.00	0.00	0.00	0.00	0.00
119	0.00	0.00	0.00	0.00	0.00	0.00
120	0.00	0.00	0.00	0.00	0.00	0.00
121	0.00	0.00	0.00	0.00	0.00	0.00
122	0.00	0.00	0.00	0.00	0.00	0.00
123	0.00	0.00	0.00	0.00	0.00	0.00
124	0.00	0.00	0.00	0.00	0.00	0.00
125	0.00	0.00	0.00	0.00	0.00	0.00
126	0.00	0.00	0.00	0.00	0.00	0.00
127	0.00	0.00	0.00	0.00	0.00	0.00
128	0.00	0.00	0.00	0.00	0.00	0.00
129	0.00	0.00	0.00	0.00	0.00	0.00
130	0.00	0.00	0.00	0.00	0.00	-0.01
131	0.00	0.00	0.00	0.00	0.00	-0.03
132	0.00	0.00	0.00	0.00	0.00	-0.07
133	0.00	0.00	0.00	0.00	0.00	-0.01
134	0.00	0.00	0.00	0.00	0.00	0.00
135	0.00	0.00	0.00	0.00	0.00	-0.01
136	0.00	0.00	0.00	0.00	0.00	-0.01
137	0.00	0.00	0.00	0.00	0.00	0.00
138	0.00	0.00	0.00	0.00	0.00	0.00
139	0.00	0.00	0.00	0.00	0.00	0.00
140	0.00	0.00	0.00	0.00	0.00	0.00
141	0.00	0.00	0.00	0.00	0.00	0.00
142	0.00	0.00	0.00	0.00	0.00	0.00
143	0.00	0.00	0.00	0.00	0.00	0.00
144	0.00	0.00	0.00	0.00	0.00	0.00
145	0.00	0.00	0.00	0.00	0.00	0.00

146	0.00	0.00	0.00	0.00	0.00	0.00
147	0.00	0.00	0.00	0.00	0.00	-0.01
148	-0.01	-0.01	-0.01	0.00	0.00	-0.02
149	0.00	0.00	0.00	0.00	0.00	-0.01
150	-0.01	-0.01	-0.01	-0.01	-0.01	-0.02
151	0.00	0.00	0.00	0.00	0.00	-0.01
152	-0.01	-0.01	-0.01	-0.01	-0.01	-0.01
153	-0.01	0.00	-0.01	0.00	-0.01	-0.01
154	0.00	0.00	0.00	0.00	0.00	0.00
155	-0.01	0.00	-0.01	0.00	-0.01	-0.01
156	-0.01	0.00	0.00	0.00	-0.01	-0.01
157	0.00	0.00	0.00	0.00	0.00	0.00
158	0.00	0.00	0.00	0.00	0.00	0.00
159	0.00	0.00	0.00	0.00	-0.01	-0.02
160	0.00	0.00	0.00	0.00	0.00	0.00
161	0.00	0.00	0.00	0.00	0.00	0.00
162	0.00	0.00	0.00	0.00	0.00	-0.01
163	0.00	0.00	0.00	0.00	0.00	0.00
164	0.00	0.00	0.00	0.00	0.00	0.00
165	0.00	0.00	0.00	0.00	0.00	0.00
166	0.00	0.00	0.00	0.00	0.00	0.00
167	0.00	0.00	0.00	0.00	0.00	0.00
168	0.00	0.00	0.00	0.00	0.00	0.00
169	0.00	0.00	0.00	0.00	0.00	0.00
170	0.00	0.00	0.00	0.00	0.00	0.00
171	0.00	0.00	0.00	0.00	0.00	0.00
172	0.00	0.00	0.00	0.00	0.00	0.00
173	0.00	0.00	0.00	0.00	0.00	0.00
174	0.00	0.00	0.00	0.00	0.00	0.00
175	0.00	0.00	0.00	0.00	0.00	0.00
176	0.00	0.00	0.00	0.00	0.00	0.00
177	0.00	0.00	0.00	0.00	0.00	0.00
178	0.00	0.00	0.00	0.00	0.00	0.00
179	0.00	0.00	0.00	0.00	0.00	0.00
180	0.00	0.00	0.00	0.00	0.00	0.00
181	0.00	0.00	0.00	0.00	0.00	0.00
182	0.00	0.00	0.00	0.00	0.00	0.00
183	0.00	0.00	0.00	0.00	0.00	0.00
184	0.00	0.00	0.00	0.00	0.00	0.00
185	0.00	0.00	0.00	0.00	0.00	0.00
186	0.00	0.00	0.00	0.00	0.00	0.00
187	0.00	0.00	0.00	0.00	0.00	0.00
188	0.00	0.00	0.00	0.00	0.00	0.00
189	0.00	0.00	0.00	0.00	0.00	0.00
190	0.00	0.00	0.00	0.00	0.00	0.00
191	0.00	0.00	0.00	0.00	0.00	0.00
192	0.00	0.00	0.00	0.00	0.00	0.00
193	0.00	0.00	0.00	0.00	0.00	0.00
194	0.00	0.00	0.00	0.00	0.00	0.00
195	0.00	0.00	0.00	0.00	0.00	-0.01

196	0.00	-0.01	-0.01	0.00	0.00	-0.01
197	0.00	0.00	-0.01	0.00	0.00	-0.01
198	0.00	0.00	-0.01	0.00	0.00	-0.01
199	0.00	-0.01	-0.02	0.00	0.00	-0.02
200	0.00	-0.02	-0.04	0.00	0.00	-0.04
201	0.00	-0.01	-0.05	0.00	0.00	-0.06
202	0.00	-0.02	-0.08	0.00	0.00	-0.12
203	-0.01	-0.18	-0.43	-0.01	-0.01	-0.46
204	-0.01	-0.34	-1.45	0.00	0.00	-1.14
205	0.00	-0.03	-0.66	0.00	0.00	-1.29
206	0.00	-0.03	-0.37	0.00	0.00	-2.18
207	0.00	-0.05	-0.72	0.00	0.00	-1.09
208	-0.02	-0.21	-0.13	-0.01	-0.01	-0.19
209	-0.01	-0.17	-0.07	-0.01	-0.02	-0.75
210	0.00	-0.01	-0.02	0.00	0.00	-0.09
211	-0.01	-0.03	-0.04	-0.01	-0.01	-0.06
212	-0.02	-0.10	-0.09	-0.02	-0.02	-0.13
213	-0.01	-0.01	-0.01	-0.01	-0.01	-0.02
214	-0.01	-0.01	-0.01	-0.01	-0.01	-0.02
215	-0.01	-0.01	-0.01	-0.01	-0.01	-0.01
216	-0.02	-0.02	-0.04	-0.02	-0.02	-0.02
217	0.00	-0.01	-0.01	-0.01	-0.01	-0.01
218	-0.01	-0.01	-0.01	-0.01	-0.01	-0.02
219	-0.02	-0.02	-0.02	-0.02	-0.02	-0.03
220	-0.01	-0.02	-0.02	-0.02	-0.03	-0.02
221	-0.01	-0.01	-0.01	-0.01	-0.02	-0.04
222	-0.01	-0.01	-0.01	-0.01	-0.02	-0.05
223	-0.04	-0.02	-0.03	-0.06	-0.08	-0.08
224	-0.03	-0.03	-0.04	-0.07	-0.21	-0.18
225	-0.36	-0.09	-0.08	-1.08	-2.25	-2.09
226	-0.30	-0.17	-0.17	-0.62	-0.58	-0.13
227	-1.40	-1.77	-1.35	-2.11	-1.31	-0.58
228	-0.81	-1.10	-2.27	-0.52	-0.40	-0.12
229	-0.70	-1.05	-2.03	-0.86	-0.28	-0.24
230	-1.22	-0.25	-0.35	-1.18	-0.09	-0.70
231	-0.07	-0.11	-0.15	-0.09	-0.08	-0.07
232	-0.08	-0.13	-0.77	-0.08	-0.09	-0.13
233	-0.86	-1.31	-0.75	-0.52	-1.05	-1.03
234	-0.15	-0.09	-0.10	-0.11	-0.12	-0.15
235	-0.04	-0.04	-0.07	-0.03	-0.04	-0.06
236	-0.19	-0.19	-0.35	-0.05	-0.26	-0.24
237	0.01	-0.18	-0.37	-0.25	-0.14	-0.16
238	-0.04	-0.04	-0.06	-0.03	-0.03	-0.06
239	-0.03	-0.18	-0.09	-0.02	-0.02	-0.06
240	-0.62	-0.79	-1.40	-0.28	-0.32	-0.41
241	-0.06	-0.09	-0.15	-0.07	-0.04	-0.11
242	-0.02	-0.04	-0.05	-0.01	-0.01	-0.04
243	-0.03	-0.57	-0.30	-0.01	-0.01	-0.13
244	-0.16	-0.70	-0.91	-0.13	-0.07	-0.59
245	-0.01	-0.02	-0.03	-0.01	-0.01	-0.03

246	-0.01	-0.06	-0.04	0.00	0.00	-0.07
247	-0.03	-0.88	-0.73	-0.01	-0.01	-1.57
248	-0.01	-0.03	-0.02	0.00	0.00	-0.05
249	-0.08	-0.12	-0.26	-0.03	-0.02	-0.16
250	-0.02	-0.02	-0.03	-0.01	-0.01	-0.04
251	-0.07	-0.05	-0.07	-0.04	-0.05	-0.12
252	-0.82	-0.28	0.22	0.24	-0.33	-0.35
253	-0.06	-0.07	-0.09	-0.04	-0.04	-0.13
254	-0.05	-0.04	-0.08	-0.05	-0.05	-0.08
255	-0.99	-0.40	0.26	0.12	0.02	-0.97
256	-0.53	-0.44	-1.07	-0.36	0.06	-0.92
257	-0.10	-0.06	-0.12	-0.11	-0.08	-0.11
258	-0.15	-0.16	-0.62	-0.58	-0.16	-0.20
259	-0.53	-1.41	0.40	-0.35	-0.64	-1.72
260	-0.26	-0.10	-0.12	-0.19	-0.19	-0.30
261	-0.12	-0.03	-0.05	-0.05	-0.07	-0.09
262	-1.27	-0.26	-0.47	-0.54	-0.97	-1.04
263	-1.97	-1.92	-0.67	-1.06	-2.16	-3.08
264	-0.15	-0.07	-0.07	-0.10	-0.16	-0.20
265	-1.23	-0.05	-0.14	-0.14	-0.24	-0.55
266	-2.09	-0.98	-0.72	-0.93	-2.78	-3.24
267	-0.71	-1.41	-1.17	-1.71	-1.84	-2.41
268	-1.43	-0.09	-0.36	-0.31	-0.66	-1.80
269	-1.32	-0.30	-0.93	-1.92	-1.99	-1.81
270	-1.75	-0.49	-0.73	-2.88	-1.39	-1.21
271	-0.33	-0.11	-0.14	-0.52	-0.27	-0.25
272	-0.10	-0.02	-0.03	-0.07	-0.11	-0.17
273	-0.07	-0.01	-0.02	-0.03	-0.25	-0.51
274	-0.04	-0.01	-0.01	-0.02	-0.06	-0.42
275	-0.09	-0.01	-0.03	-0.03	-0.08	-0.17
276	-0.46	-0.01	-0.06	-0.02	-0.32	-1.29
277	-0.19	0.00	-0.01	0.00	-0.02	-0.10
278	-0.12	0.00	-0.01	-0.01	-0.02	-0.06
279	-1.05	-0.01	-0.04	-0.02	-0.08	-0.49
280	-1.78	0.00	-0.04	-0.01	-0.06	-0.64
281	-0.26	0.00	-0.01	-0.01	-0.05	-0.11
282	-0.07	0.00	-0.02	-0.01	-0.23	-0.07
283	-0.06	-0.07	-0.56	-0.50	-1.27	-0.17
284	-0.02	-0.01	-0.04	-0.03	-0.48	-0.04
285	-0.02	0.00	-0.01	-0.01	-0.05	-0.03
286	-0.15	-0.01	-0.04	-0.03	-0.12	-0.15
287	-0.02	-0.01	-0.02	-0.02	-0.03	-0.02
288	-0.01	0.00	-0.01	-0.01	-0.01	-0.01
289	-0.02	0.00	-0.01	0.00	-0.01	-0.02
290	-0.02	-0.01	-0.02	-0.02	-0.03	-0.04
291	0.00	0.00	0.00	-0.01	-0.01	-0.01
292	-0.01	0.00	0.00	0.00	-0.01	-0.01
293	-0.01	0.00	-0.01	-0.01	-0.01	-0.04
294	-0.01	0.00	0.00	0.00	-0.01	-0.40
295	0.00	0.00	0.00	-0.01	-0.02	-0.68

296	-0.02	-0.01	-0.01	-0.04	-0.10	-0.50
297	-0.01	-0.01	-0.01	-0.03	-0.38	-2.05
298	-0.01	-0.02	-0.02	-0.05	-0.50	-2.32
299	-0.03	-0.07	-0.05	-0.48	-1.28	-2.00
300	-0.01	-0.05	-0.15	-0.33	-1.07	-2.67
301	-0.01	-0.78	-0.28	-0.29	-1.10	-2.81
302	-0.04	-0.77	-0.13	-0.28	-1.12	-1.83
303	-0.22	-2.48	-0.23	-0.67	-0.90	-1.30
304	-0.05	-1.41	-0.12	-0.17	-0.12	-0.24
305	-0.06	-0.21	-0.10	-0.21	-0.11	-0.05
306	-0.03	-0.95	-0.09	-0.07	-0.03	-0.04
307	-0.18	-0.99	-0.89	-0.44	-0.10	-0.32
308	-1.08	-0.35	-0.29	-1.29	-0.23	-0.10
309	-0.55	-0.18	-0.30	-1.04	-1.76	-0.04
310	-0.04	-0.86	-0.19	-0.05	-0.16	-0.02
311	-0.04	-0.59	-1.38	-0.12	-0.98	-0.02
312	-0.67	-0.20	-1.18	-0.44	-1.02	-0.04
313	-0.10	-0.71	-1.17	-0.56	-0.78	-0.06
314	-0.44	-0.34	-0.60	-1.54	-1.24	-0.26
315	-0.33	-0.11	-0.14	-0.27	-0.17	-0.07
316	-0.04	-0.59	-0.05	-0.11	-0.10	-0.33
317	-0.30	-1.13	-0.49	-2.25	-1.78	-1.83
318	-1.85	-0.25	-1.66	-2.61	-1.20	-0.73
319	-0.09	-0.90	-0.15	-0.16	-0.17	-0.59
320	-0.05	-0.77	-0.24	-0.21	-0.42	-1.99
321	-0.47	-0.91	-0.95	-1.83	-2.58	-3.22
322	-0.66	-0.20	-0.46	-0.91	-0.49	-1.59
323	-0.07	-0.37	-0.13	-0.10	-0.18	-1.98
324	-0.04	-0.08	-0.09	-0.07	-0.04	-1.49
325	-0.77	-0.12	-1.15	-1.23	-0.12	-1.02
326	-1.12	-0.11	-0.78	-1.70	-0.36	-0.16
327	-0.06	-0.05	-0.06	-0.09	-0.03	-0.08
328	-0.04	-0.02	-0.07	-0.05	-0.02	-0.03
329	-1.43	-0.64	-2.09	-1.86	-0.98	-0.06
330	-0.06	-0.06	-0.08	-0.13	-0.10	-0.02
331	-0.02	-0.03	-0.03	-0.03	-0.03	-0.02
332	-0.13	-1.15	-0.78	-0.33	-0.61	-0.07
333	-0.05	0.20	-0.35	-0.52	-0.50	-0.05
334	-0.01	-0.04	-0.03	-0.03	-0.04	-0.02
335	-0.01	-0.01	-0.01	-0.01	-0.02	-0.02
336	-0.11	-0.48	-0.84	-0.30	-0.65	-0.29
337	-0.05	-0.10	-0.07	-0.17	-0.18	-0.17
338	0.00	-0.01	-0.01	-0.01	-0.02	-0.11
339	0.00	-0.01	-0.01	-0.01	-0.02	-0.14
340	0.00	0.00	0.00	0.00	-0.01	-0.20
341	0.00	-0.01	0.00	0.00	-0.01	-0.04
342	0.00	0.00	0.00	0.00	0.00	-0.01
343	0.00	0.00	0.00	0.00	0.00	-0.01
344	0.00	-0.01	0.00	0.00	0.00	-0.01
345	0.00	-0.01	0.00	0.00	0.00	-0.01

346	0.00	-0.01	-0.01	-0.01	-0.01	-0.01
347	0.00	-0.02	-0.01	0.00	0.00	-0.01
348	0.00	-0.08	0.00	0.00	0.00	-0.01
349	0.00	-0.06	0.00	0.00	-0.01	-0.01
350	0.00	-0.03	-0.01	-0.01	0.00	-0.01
351	0.00	-0.22	-0.01	0.00	0.00	-0.10
352	0.00	-1.26	0.00	0.00	0.00	-0.01
353	-0.03	-0.19	-0.03	-0.04	-0.05	-0.03
354	-0.01	-0.39	-0.01	-0.01	-0.01	-0.19
355	0.00	-2.36	0.00	0.00	0.00	-0.12
356	0.00	-2.20	-0.01	0.00	-0.01	-0.02
357	-0.02	-0.33	-0.02	-0.03	-0.02	-0.06
358	0.00	-3.43	-0.01	-0.01	-0.01	-0.16
359	0.00	-2.44	0.00	0.00	0.00	-0.07
360	0.00	-0.28	-0.01	-0.01	-0.01	-0.04
361	-0.01	-1.27	-0.01	-0.01	-0.02	-0.35
362	0.00	-1.41	0.00	0.00	0.00	-0.42
363	0.00	-0.58	0.00	0.00	-0.01	-0.16
364	0.00	-1.42	-0.01	0.00	-0.01	-0.20
365	0.00	-1.87	-0.04	0.00	-0.02	-0.05
366	0.00	-0.90	-0.02	-0.01	-0.02	-0.17
367	-0.01	-1.74	-0.69	-0.03	-0.08	-0.02
368	0.00	-0.28	-0.06	0.00	-0.01	-0.01
369	0.00	-0.26	-0.01	0.00	0.00	-0.01
370	-0.02	-0.22	-0.06	-0.03	-0.05	-0.03
371	0.00	-0.29	-0.07	0.00	-0.01	0.00
372	0.00	-1.37	-0.01	0.00	0.00	0.00
373	0.00	-0.58	-0.01	0.00	-0.01	-0.01
374	-0.01	-0.16	-0.02	-0.01	-0.03	-0.01
375	0.00	-0.03	0.00	0.00	0.00	0.00
376	0.00	-1.11	0.00	0.00	0.00	0.00
377	0.00	-0.18	-0.01	0.00	-0.01	-0.01
378	0.00	-0.06	0.00	0.00	0.00	0.00
379	0.00	-0.20	0.00	0.00	0.00	0.00
380	0.00	-0.48	0.00	0.00	0.00	0.00
381	0.00	-0.01	0.00	0.00	0.00	-0.01
382	0.00	-0.01	0.00	0.00	0.00	0.00
383	0.00	-0.02	0.00	0.00	0.00	0.00
384	0.00	0.00	0.00	0.00	0.00	0.00
385	0.00	0.00	0.00	0.00	0.00	0.00
386	0.00	0.00	0.00	0.00	0.00	0.00
387	0.00	0.00	0.00	0.00	0.00	0.00
388	0.00	0.00	0.00	0.00	0.00	0.00
389	0.00	0.00	0.00	0.00	0.00	0.00
390	0.00	0.00	0.00	0.00	0.00	0.00
391	0.00	0.00	0.00	0.00	0.00	0.00
392	0.00	0.00	0.00	0.00	0.00	0.00
393	0.00	0.00	0.00	0.00	0.00	0.00
394	0.00	0.00	0.00	0.00	0.00	0.00
395	0.00	0.00	0.00	0.00	0.00	0.00

396	0.00	0.00	0.00	0.00	0.00	0.00
397	0.00	0.00	0.00	0.00	0.00	0.00
398	0.00	0.00	0.00	0.00	0.00	0.00
399	0.00	0.00	0.00	0.00	0.00	0.00
400	0.00	0.00	0.00	0.00	0.00	0.00
401	0.00	0.00	0.00	0.00	0.00	0.00
402	0.00	0.00	0.00	0.00	0.00	0.00
403	0.00	0.00	0.00	0.00	0.00	0.00
404	0.00	0.00	0.00	0.00	0.00	0.00
405	0.00	0.00	0.00	0.00	0.00	0.00
406	0.00	0.00	0.00	0.00	0.00	0.00
407	0.00	0.00	0.00	0.00	0.00	0.00
408	0.00	0.00	0.00	0.00	0.00	0.00
409	0.00	0.00	0.00	0.00	0.00	0.00
410	0.00	0.00	0.00	0.00	0.00	0.00
411	0.00	0.00	0.00	0.00	0.00	0.00
412	0.00	0.00	0.00	0.00	0.00	0.00
413	0.00	0.00	0.00	0.00	0.00	0.00
414	0.00	0.00	0.00	0.00	0.00	0.00
415	0.00	0.00	0.00	0.00	0.00	0.00
416	0.00	0.00	0.00	0.00	0.00	0.00
417	0.00	0.00	0.00	0.00	0.00	0.00
418	0.00	0.00	0.00	0.00	0.00	0.00
419	0.00	0.00	0.00	0.00	0.00	0.00
420	0.00	0.00	0.00	0.00	0.00	0.00
421	0.00	0.00	0.00	0.00	0.00	0.00
422	0.00	0.00	0.00	0.00	0.00	0.00
423	0.00	0.00	0.00	0.00	0.00	0.00
424	0.00	0.00	0.00	0.00	0.00	0.00
425	0.00	0.00	0.00	0.00	0.00	0.00
426	0.00	0.00	0.00	0.00	0.00	0.00
427	0.00	0.00	0.00	0.00	0.00	0.00
428	0.00	0.00	0.00	0.00	0.00	0.00
429	0.00	0.00	0.00	0.00	0.00	0.00
430	0.00	0.00	0.00	0.00	0.00	0.00
431	0.00	0.00	0.00	0.00	0.00	0.00
432	0.00	0.00	0.00	0.00	0.00	0.00
433	0.00	0.00	0.00	0.00	0.00	0.00
434	0.00	0.00	0.00	0.00	0.00	0.00
435	0.00	0.00	0.00	0.00	0.00	0.00
436	0.00	0.00	0.00	0.00	0.00	0.00
437	0.00	0.00	0.00	0.00	0.00	0.00
438	0.00	0.00	0.00	0.00	0.00	0.00
439	0.00	0.00	0.00	0.00	0.00	0.00
440	0.00	0.00	0.00	0.00	0.00	-0.01
441	0.00	0.00	0.00	0.00	0.00	-0.04
442	0.00	0.00	0.00	0.00	0.00	0.00
443	0.00	0.00	0.00	0.00	0.00	-0.12
444	0.00	0.00	0.00	0.00	0.00	-0.07
445	0.00	0.00	0.00	0.00	0.00	-0.01

446	0.00	0.00	0.00	0.00	0.00	-0.01
447	0.00	0.00	0.00	0.00	0.00	-0.01
448	0.00	0.00	0.00	0.00	0.00	0.00
449	0.00	0.00	0.00	0.00	0.00	0.00
450	0.00	0.00	0.00	0.00	0.00	0.00
451	0.00	0.00	0.00	0.00	0.00	0.00
452	0.00	0.00	0.00	0.00	0.00	0.00
453	0.00	0.00	0.00	0.00	0.00	0.00
454	0.00	0.00	0.00	0.00	0.00	0.00
455	0.00	0.00	0.00	0.00	0.00	0.00
456	0.00	0.00	0.00	0.00	0.00	0.00
457	0.00	0.00	0.00	0.00	0.00	0.00
458	0.00	0.00	0.00	0.00	0.00	0.00
459	0.00	0.00	0.00	0.00	0.00	0.00
460	0.00	0.00	0.00	0.00	0.00	0.00
461	0.00	0.00	0.00	0.00	0.00	-0.01
462	0.00	0.00	0.00	0.00	0.00	0.00
463	0.00	0.00	0.00	0.00	0.00	0.00
464	0.00	0.00	0.00	0.00	0.00	-0.01
465	0.00	0.00	0.00	0.00	0.00	-0.02
466	0.00	0.00	0.00	0.00	0.00	0.00
467	0.00	0.00	0.00	0.00	0.00	0.00
468	0.00	0.00	0.00	0.00	0.00	0.00
469	0.00	0.00	0.00	0.00	0.00	0.00
470	0.00	0.00	0.00	0.00	0.00	0.00
471	0.00	0.00	0.00	0.00	0.00	-0.01
472	0.00	0.00	0.00	0.00	0.00	0.00
473	0.00	0.00	0.00	0.00	0.00	-0.01
474	0.00	0.00	0.00	0.00	0.00	-0.04
475	0.00	-0.01	0.00	0.00	0.00	-0.06
476	0.00	0.00	0.00	0.00	0.00	-0.01
477	0.00	0.00	0.00	0.00	0.00	-0.04
478	0.00	-0.01	0.00	0.00	0.00	-0.35
479	0.00	-0.03	0.00	0.00	0.00	-0.52
480	0.00	-0.01	0.00	0.00	0.00	-0.08
481	0.00	-0.01	0.00	0.00	0.00	-0.06
482	0.00	-0.02	0.00	0.00	0.00	-0.02
483	0.00	-0.02	0.00	0.00	0.00	-0.01
484	0.00	-0.01	0.00	0.00	0.00	-0.02
485	0.00	-0.01	0.00	0.00	0.00	0.00
486	0.00	-0.01	0.00	0.00	0.00	0.00
487	0.00	0.00	0.00	0.00	0.00	0.00
488	0.00	0.00	0.00	0.00	0.00	0.00
489	0.00	0.00	0.00	0.00	0.00	0.00
490	0.00	0.00	0.00	0.00	0.00	0.00
491	0.00	0.00	0.00	0.00	0.00	0.00
492	0.00	0.00	0.00	0.00	0.00	0.00
493	0.00	0.00	0.00	0.00	0.00	0.00
494	0.00	0.00	0.00	0.00	0.00	0.00
495	0.00	0.00	0.00	0.00	0.00	0.00

496	0.00	0.00	0.00	0.00	0.00	0.00
497	0.00	0.00	0.00	0.00	0.00	0.00
498	0.00	0.00	0.00	0.00	0.00	0.00
499	0.00	0.00	0.00	0.00	0.00	0.00
500	0.00	0.00	0.00	0.00	0.00	0.00
501	0.00	0.00	0.00	0.00	0.00	0.00
502	0.00	0.00	0.00	0.00	0.00	0.00
503	0.00	0.00	0.00	0.00	0.00	0.00
504	0.00	0.00	0.00	0.00	0.00	0.00
505	0.00	0.00	0.00	0.00	0.00	0.00
506	0.00	0.00	0.00	0.00	0.00	0.00
507	0.00	0.00	0.00	0.00	0.00	0.00
508	0.00	0.00	0.00	0.00	0.00	0.00
509	0.00	0.00	0.00	0.00	0.00	0.00
510	0.00	0.00	0.00	0.00	0.00	0.00
511	0.00	0.00	0.00	0.00	0.00	0.00
512	0.00	0.00	0.00	0.00	0.00	0.00
513	0.00	0.00	0.00	0.00	0.00	0.00
514	0.00	0.00	0.00	0.00	0.00	0.00
515	0.00	0.00	0.00	0.00	0.00	0.00
516	0.00	0.00	0.00	0.00	0.00	0.00
517	0.00	0.00	0.00	0.00	0.00	0.00
518	0.00	0.00	0.00	0.00	0.00	0.00
519	0.00	0.00	0.00	0.00	0.00	0.00
520	0.00	0.00	0.00	0.00	0.00	0.00
521	0.00	0.00	0.00	0.00	0.00	0.00
522	0.00	0.00	0.00	0.00	0.00	0.00
523	0.00	0.00	0.00	0.00	0.00	0.00
524	0.00	0.00	0.00	0.00	0.00	0.00
525	0.00	0.00	0.00	0.00	0.00	0.00
526	0.00	0.00	0.00	0.00	0.00	0.00
527	0.00	0.00	0.00	0.00	0.00	0.00
528	0.00	0.00	0.00	0.00	0.00	0.00
529	0.00	0.00	0.00	0.00	0.00	0.00
530	0.00	0.00	0.00	0.00	0.00	0.00
531	0.00	0.00	0.00	0.00	0.00	0.00
532	0.00	0.00	0.00	0.00	0.00	0.00
533	0.00	0.00	0.00	0.00	0.00	0.00
534	0.00	0.00	0.00	0.00	0.00	0.00
535	0.00	0.00	0.00	0.00	0.00	0.00
536	0.00	0.00	0.00	0.00	0.00	0.00
537	0.00	0.00	0.00	0.00	0.00	0.00
538	0.00	0.00	0.00	0.00	0.00	0.00
539	0.00	0.00	0.00	0.00	0.00	0.00
540	0.00	0.00	0.00	0.00	0.00	0.00
541	0.00	0.00	0.00	0.00	0.00	0.00
542	0.00	0.00	0.00	0.00	0.00	0.00
543	0.00	0.00	0.00	0.00	0.00	0.00
544	0.00	0.00	0.00	0.00	0.00	0.00
545	0.00	0.00	0.00	0.00	0.00	0.00

546	0.00	0.00	0.00	0.00	0.00	0.00
547	0.00	0.00	0.00	0.00	0.00	0.00
548	0.00	0.00	0.00	0.00	0.00	0.00
549	0.00	0.00	0.00	0.00	0.00	0.00
550	0.00	0.00	0.00	0.00	0.00	0.00
551	0.00	0.00	0.00	0.00	0.00	0.00
552	0.00	0.00	0.00	0.00	0.00	0.00
553	0.00	0.00	0.00	0.00	0.00	0.00
554	0.00	0.00	0.00	0.00	0.00	0.00
555	0.00	0.00	0.00	0.00	0.00	0.00
556	0.00	0.00	0.00	0.00	0.00	0.00
557	0.00	0.00	0.00	0.00	0.00	0.00
558	0.00	0.00	0.00	0.00	0.00	0.00
559	0.00	0.00	0.00	0.00	0.00	0.00
560	0.00	0.00	0.00	0.00	0.00	0.00
561	0.00	0.00	0.00	0.00	0.00	0.00
562	0.00	0.00	0.00	0.00	0.00	0.00
563	0.00	0.00	0.00	0.00	0.00	0.00
564	0.00	0.00	0.00	0.00	0.00	0.00
565	0.00	0.00	0.00	0.00	0.00	0.00
566	0.00	0.00	0.00	0.00	0.00	0.00
567	0.00	0.00	0.00	0.00	0.00	0.00
568	0.00	0.00	0.00	0.00	0.00	0.00
569	0.00	0.00	0.00	0.00	0.00	0.00
570	0.00	0.00	0.00	0.00	0.00	0.00
571	0.00	0.00	0.00	0.00	0.00	0.00
572	0.00	0.00	0.00	0.00	0.00	0.00
573	0.00	0.00	0.00	0.00	0.00	0.00
574	0.00	0.00	0.00	0.00	0.00	0.00
575	0.00	0.00	0.00	0.00	0.00	0.00
576	0.00	0.00	0.00	0.00	0.00	0.00
577	0.00	0.00	0.00	0.00	0.00	0.00
578	0.00	0.00	0.00	0.00	0.00	0.00
579	0.00	0.00	0.00	0.00	0.00	0.00
580	0.00	0.00	0.00	0.00	0.00	0.00
581	0.00	0.00	0.00	0.00	0.00	0.00
582	0.00	0.00	0.00	0.00	0.00	0.00
583	0.00	0.00	0.00	0.00	0.00	0.00
584	0.00	0.00	0.00	0.00	0.00	0.00
585	0.00	0.00	0.00	0.00	0.00	0.00
